# Genetic and biological insights into *Hydatigera taeniaeformis* in invasive black rats from southern Chile

**DOI:** 10.3389/fvets.2024.1466409

**Published:** 2024-11-13

**Authors:** Cristian A. Alvarez Rojas, Cristian Bonacic, Rodrigo Salgado, Lucia Peters, Diego Fredes, André V. Rubio, Sebastián Muñoz-Leal, Pablo Oyarzún-Ruiz, A. Alonso Aguirre

**Affiliations:** ^1^Escuela de Medicina Veterinaria, Facultad de Agronomía y Sistemas Naturales, Facultad de Ciencias Biológicas y Facultad de Medicina, Pontificia Universidad Católica de Chile, Santiago, Chile; ^2^Laboratorio Fauna Australis, Facultad de Agronomía y Sistemas Naturales, Pontificia Universidad Católica de Chile, Santiago, Chile; ^3^Departamento de Ciencias Biológicas Animales, Facultad de Ciencias Veterinarias y Pecuarias, Universidad de Chile, Santiago, Chile; ^4^Departamento de Ciencia Animal, Facultad de Ciencias Veterinarias, Universidad de Concepción, Chillán, Chile; ^5^Departamento de Microbiología, Facultad de Ciencias Veterinarias, Universidad de Concepción, Chillán, Chile; ^6^Department of Fish, Wildlife and Conservation Biology, Warner College Natural Resources, Colorado State University, Fort Collins, CO, United States

**Keywords:** Chile, *cox1* gene, genetic variability, *Hydatigera taeniaeformis*, *Rattus rattus*

## Abstract

**Introduction:**

This study investigates the genetic variability of *Hydatigera taeniaeformis* in black rats (*Rattus rattus*), a common tapeworm that infects cats and rodents worldwide. Despite its widespread presence and zoonotic potential, little is known about the genetic diversity of this parasite in the Americas.

**Methods:**

We conducted DNA barcoding analysis using mitochondrial *cox*1 gene sequences using samples collected from 171 invasive wild black rats, captured in the temperate rainforest of Southern Chile. We also included two adult parasites isolated from road killed Kodkods (*Leopardus guigna*), a small felid species native to the Americas.

**Results:**

Our findings revealed only two haplotypes, suggesting low genetic variability in a parasite that arrived in the Americas with the Spanish colonization.

**Discussion:**

These haplotypes are more closely related to parasite populations from Peru, Africa, Australia, and Europe, suggesting an origin linked to the Spanish colonization, possibly from North Africa via the Canary Islands. The study also analyzed infection rates, parasite size, and their correlation with host body size, age, and weight, revealing significant patterns. These results provide new insights into the biogeography and genetic diversity of *H*. *taeniaeformis* in a new geographical area, enhancing our understanding of its evolutionary history.

## 1 Introduction

*Hydatigera taeniaeformis* is a common and widespread tapeworm of domestic cats (*Felis catus*) and wild felids (1). Intermediate hosts are typically mice and rats. The strobilocercus-type metacestode, formerly known as *Strobilocercus fasciolaris*, develops in the liver (2). Lavikainen et al. (1), contributed to clarify the biogeography and the host spectrum of the cryptic lineages of *H*. *taeniaeformis* through DNA barcoding using partial mitochondrial cytochrome c oxidase subunit 1 (*cox*1); concluding that *H. taeniaeformis* is a complex of three morphologically cryptic entities which can be differentiated genetically, including: *H*. *taeniaeformis sensu stricto* with a worldwide distribution; *H*. *kamiyai*, mainly found in northern Eurasia; and a third lineage with unknown taxonomy found in the Mediterranean region (1). Unfortunately, no specimens from the Americas were included in this seminal study. Recently, Gomez-Puerta et al. (3) described natural infection with *H*. *taeniaeformis* in native rodents (*Oligoryzomys microtis*, *Oligoryzomys* sp. and *Simosciurus nebouxii*), from Peru.

Domestic cats arrived in the Americas during European colonization, along with infested rats (*Rattus* spp.) and mice (*Mus musculus*). Old chronicles reported that voyagers onboard the Mayflower and residents of Jamestown brought cats with them to control rats and mice and to bring good luck (4). These rodents were not only a nuisance but also posed health risks due to the diseases they could carry, such as the plague. The introduction of cats to new environments has had mixed impacts. While they helped control pest populations, they also became predators of native wildlife, impacting local bird, mammal, and reptile populations, especially on islands and in closed ecosystems. On the other hand, *R. norvegicus* and *R. rattus* spread rapidly throughout the Americas after their introduction. They disembarked from ships at ports and quickly established populations, thriving in both urban and rural settings.

Studies of genetic variability of this parasite can offer insights into the evolutionary history and biogeography. We hypothesize that this parasite shows low genetic variability in Chile due to its relatively recently arrival in South America (500 years). In the present study we performed a DNA barcoding analysis using a *cox*1 section from isolates of *H*. *taeniaeformis* from *R. rattus* collected in the southern Chile. We also analyzed biological characteristics of the host and parasites.

## 2 Materials and methods

### 2.1 Study area and rat trapping

**Figure 1:**
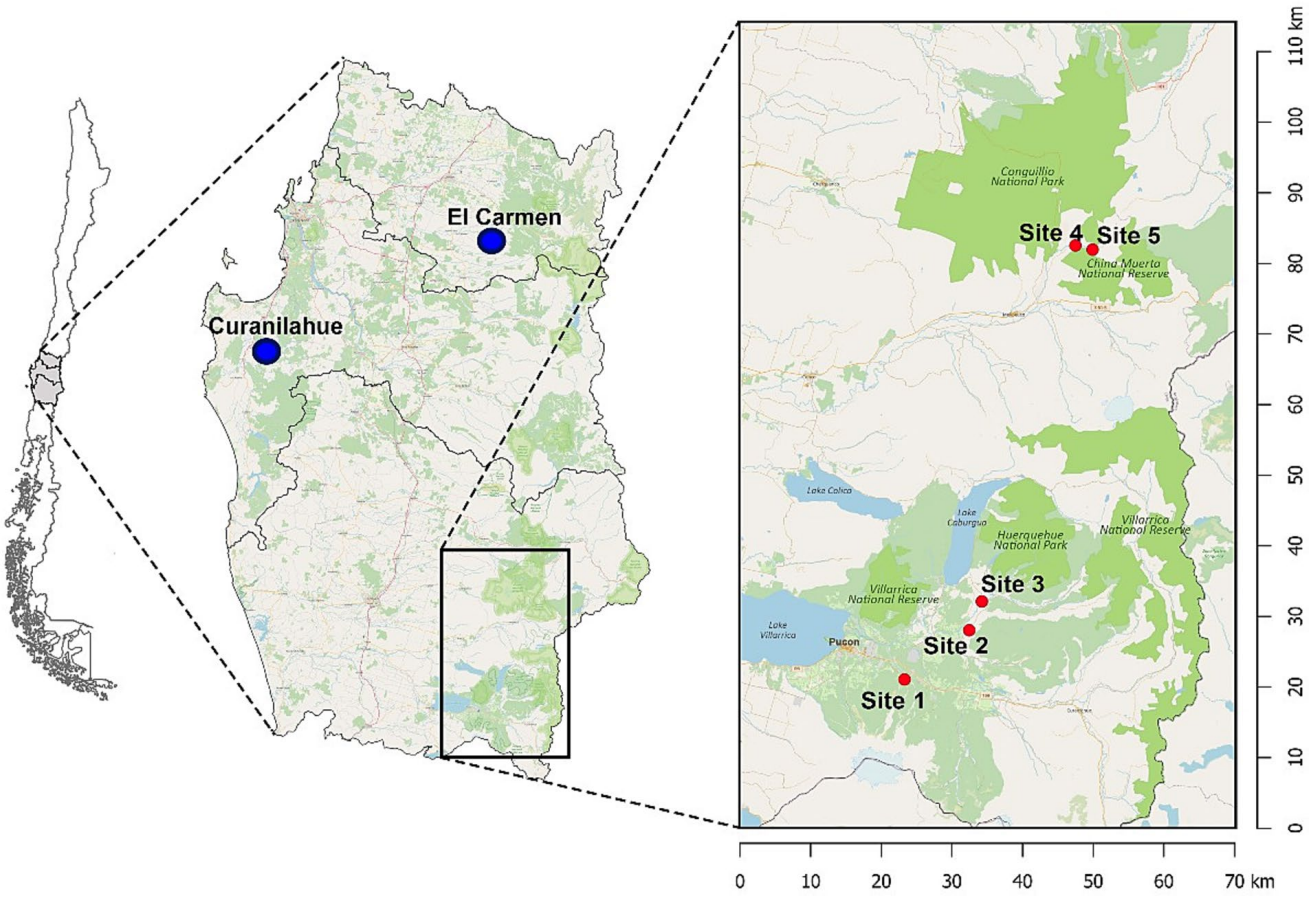
Map showing the study sites and locations of road-killed kodkods in Southern Chile. The main map highlights the temperate rainforest regions in the Araucanía and Biobío regions. The inset map zooms in on the study sites within the Araucanía region, detailing the four specific sites where black rats (*Rattus rattus*) were captured: Site 1 (Kawelluco), Site 2 (Kod Kod), Site 3 (Huife), Site 4 (China Muerta), and Site 5 (China Muerta). Blue dots indicate the locations of road-killed kodkods: El Carmen and Curanilahue. Green areas represent national parks and reserves.

This study was conducted in 2022 and 2023 across five sites in the temperate rainforest of the Araucania region, Southern Chile (39°16’S 71°48’W). Site 1 (Kawelluco), Site 2 (Kod Kod), Site 3 (Huife) and Site 4 and 5 (China Muerta) (Figure 1). The region experiences a temperate humid climate with an average annual rainfall of 2,000 mm. Rats were captured using wire mesh traps placed at 5 m intervals along transects, with a total of 50 traps per site, active for five consecutive nights per session. Traps were baited with oats and vanilla essence or peanut butter and checked daily.

### 2.2 Euthanasia and necropsy

Body weight and standard body measurements were done for each black rat captured (tail, and right foot length). Sex determination (male/female) was performed using evidence of external genitalia. Rats were euthanized using an overdose of isoflurane, and necropsies were performed to collect livers containing *H. taeniaeformis* strobilocerci, which were preserved in 70% ethanol. Rodent trapping was authorized by the Servicio Agrícola y Ganadero (SAG) under permit No. 584 (updated 23 April 2022). Bioethical approval No. 220309009 (23 April 2022) was granted by Animal Care Unit, Pontificia Universidad Católica.

### 2.3 Parasite dissection

Metacestodes were opened with scalpel blades of single usage and strobilocercus were measured in mm. The larvae exhibited a large scolex with a long neck and a pseudo-segmented body, characterized by a terminally bulged portion. A small section of each strobilocercus was excised and washed in PBS prior to DNA isolation.

### 2.4 Data analysis

Prevalences were calculated for the total number of black rats for every location, age and sex and presence of liver parasite cysts. Furthermore, the average length of the parasites was calculated following the same categories. The length and the weight of rats were then compared with the length of the parasites by generating dynamic tables with these factors and doing a correlation analysis. Linear regressions of abundance and intensity of infection were calculated following Bush et al. (5). Relative abundance of rats (captures/night; traps/site) and number of parasites present population (n° of parasites/total n° of rats) and intensity of infection (n° of parasites present in the infected animals/n° of infected rats) were calculated. Generalized linear models (GLMs) with binomial distribution and logit function were used to assess the association of prevalence with the following explanatory variables: year, location, sex and body length. The best model was based on the lowest Akaike information criterion adjusted for sample size (AICc). We performed these analyses with R software (25), including packages “MuMIn” (6).

### 2.5 Adult *Taenia* spp. from Kodkods

Proglottids of *H*. *taeniaeformis* collected from the small intestine of three Kodkods (*Leopardus guigna*) were included in the study for genetic analysis. The animals were road-killed and found in El Carmen (Ñuble region) and Curanilahue municipalities (Biobío region) in south-central Chile (Figure 1).

### 2.6 DNA extraction, amplification, and sequencing

Genomic DNA was isolated from a section of each strobilocercus and from the proglottids using the E.Z.N.A.^®^ Tissue DNA Kit following the manufacturer’s instructions. Amplification of a fragment of the mitochondrial *cox1* gene for all isolates was accomplished using the primers JB3 (5′-TTTTTTGGGCATCCTGAGGTTTAT-3′) and JB4.5 (5′-TAAAGAAAGAACATAATGAAAATG-3′) (7). The thermocycler conditions were 95°C for 2 min, and 35 cycles at 95°C for 30 s, 50°C for 30 s, and 72°C for 30 s. Five microliters of each amplicon were visualized on a 1.5% agarose gel stained with GelRedTM (Biotium, Fremont, United States) under an ultraviolet light transilluminator. After visualization, the PCR products were sequenced in both directions at Macrogen (Chile) using PCR primers. The acquired sequences were curated using the Geneious Prime 2024.03^1^ for subsequent multiple sequence alignment with all similar entries deposited in the NCBI database to investigate the intraspecific variations. The alignment was used to build a median-joining haplotype network using PopART (8).

## 3 Results

A total of 171 black rats were captured in five sites. Capture success (number of rats captured by sampling effort) was similar between years (2022–2023) from which 33 animals were infected with *H*. *taeniaeformis* (19.2%). Between Sites 1, 2 and 3, evaluated in both 2022 and 2023, a difference in prevalence was observed in 2022 (GLM, *p* = 0.012), with the highest prevalence in Site 3 (54%), but no difference was observed in 2023. Prevalence decreased from 2022 to 2023 at these sites (GLM, *p* = 0.0261). Sites 4 and 5, sampled only in 2023, showed one and zero animals positive for *H. taeniformis*, respectively. Overall, no differences in sex or body length were observed. Details of captured and positive animals in Tables 1, 2.

**Table 1:** Number of captured and infected black rats with *Hydatigera taeniaeformis* (sex and age from each location in southern Chile, 2022–2023).

Location of capture	2022		2023	
	# captured	# infected (%)	# captured	# infected (%)
Site 1 (Kawelluco)	60	18 (30%)	29	4 (13.8%)
Site 2 (Kod Kod)	25	3 (12%)	11	1 (9%)
Site 3 (Huife)	11	6 (54.5%)	4	0
Site 4 (China Muerta 1)	–	–	26	1 (3.8%)
Site 5 (China Muerta 2)	–	–	5	0
Total	96	27 (28.4%)	75	6 (8%)

**Table 2:** Number of captured and infected black rats (*Rattus rattus*) by location according to the year of capture, southern Chile, 2022–2023.

Location of capture	Total		Female		Male	
	# captured	# infected	# captured	# infected	# captured	# infected
Site 1 (Kawelluco)	89	22 (24.7%)	46	12 (26.1%)	43	10 (23.2%)
Site 2 (Kod Kod)	36	4 (10.2%)	19	4 (21.0%)	20	0
Site 3 (Huife)	15	6 (40%)	9	4 (44.4%)	6	2 (33.3%)
Site 4 (China Muerta 1)	26	1 (3.8%)	14	0	12	1 (8.3%)
Site 5 (China Muerta 2)	5	0	4	0	1	0
Total	171	33 (19.2%)	90	20 (22.2%)	81	13 (16%)

There was a positive correlation between body length and average parasite length, with a correlation coefficient of 0.40 (*p*<0.001). This indicates that animals with greater body lengths tend to have longer parasites. Secondly, the correlation between weight (in grams) and average parasite length was stronger, with a coefficient of 0.76 (*p*<0.001).

**Figure 2:**
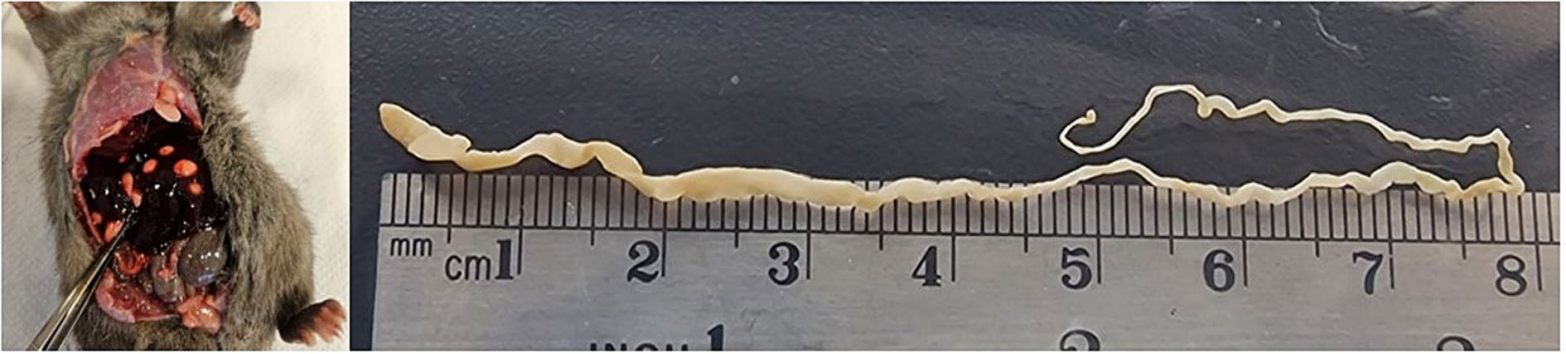
(A) Black rat (*Rattus rattus*) with strobilocerci in the liver, (B) longest strobilocercus found in the present study in the same rat.

Single strobilocerci were found in 28 rats, double infection was found in 2 and 4 strobilocerci were found in 2 animals. One animal was infected with 23 strobilocerci (Figure 2). The smallest strobilocercus measured 4 mm and the largest one 105 mm, the average length was 57.13 mm. The mean length of the cestodes was longer in Site 4 (105.1 mm), and shorter in Site 2 (22.2 mm) location. The average length of the cestodes, was longer in adults (77.6 mm), compared to sub-adults (19.00 mm) and young (10.3) rats. The average length was longer in captures from 2023 with 91.2 mm opposed to 2022 with 24 mm. The mean length of the metacestodes was 77.1 mm in males opposed to 32 mm in female rats (see Table 3).

**Table 3:** Average length and number of strobilocercus according to the year of capture, location and sex of infected black rats (*Rattus rattus*), southern Chile, 2022–2023.

Location of capture	Sex	2022		2023		Total	
		# cestodes	Length (average)	# cestodes	Length	# cestodes	Length
Site 1 (Kawelluco)	Male	7	15 mm	3	31.4 mm	10	21 mm
	Female	14	28.2 mm	4	11.8 mm	18	60.7 mm
Site 2 (Kod Kod)	Male	0	–	0	–	0	–
	Female	4	23.5 mm	1	17 mm	5	22.2 mm
Site 3 (Huife)	Male	2	37 mm	0	–	2	37 mm
	Female	5	53.2 mm	0	–	5	53.2 mm
Site 4 (China Muerta 1)	Male	–	–	23	105.1 mm	23	65 mm
	Female	–	–	0	–	0	–
Site 5 (China Muerta 2)	Male	–	–	0	–	0	–
	Female	–	–	0	–	0	–
Total		32	31.1 mm	31	83.9 mm	63	57.1 mm

**Figure 3:**
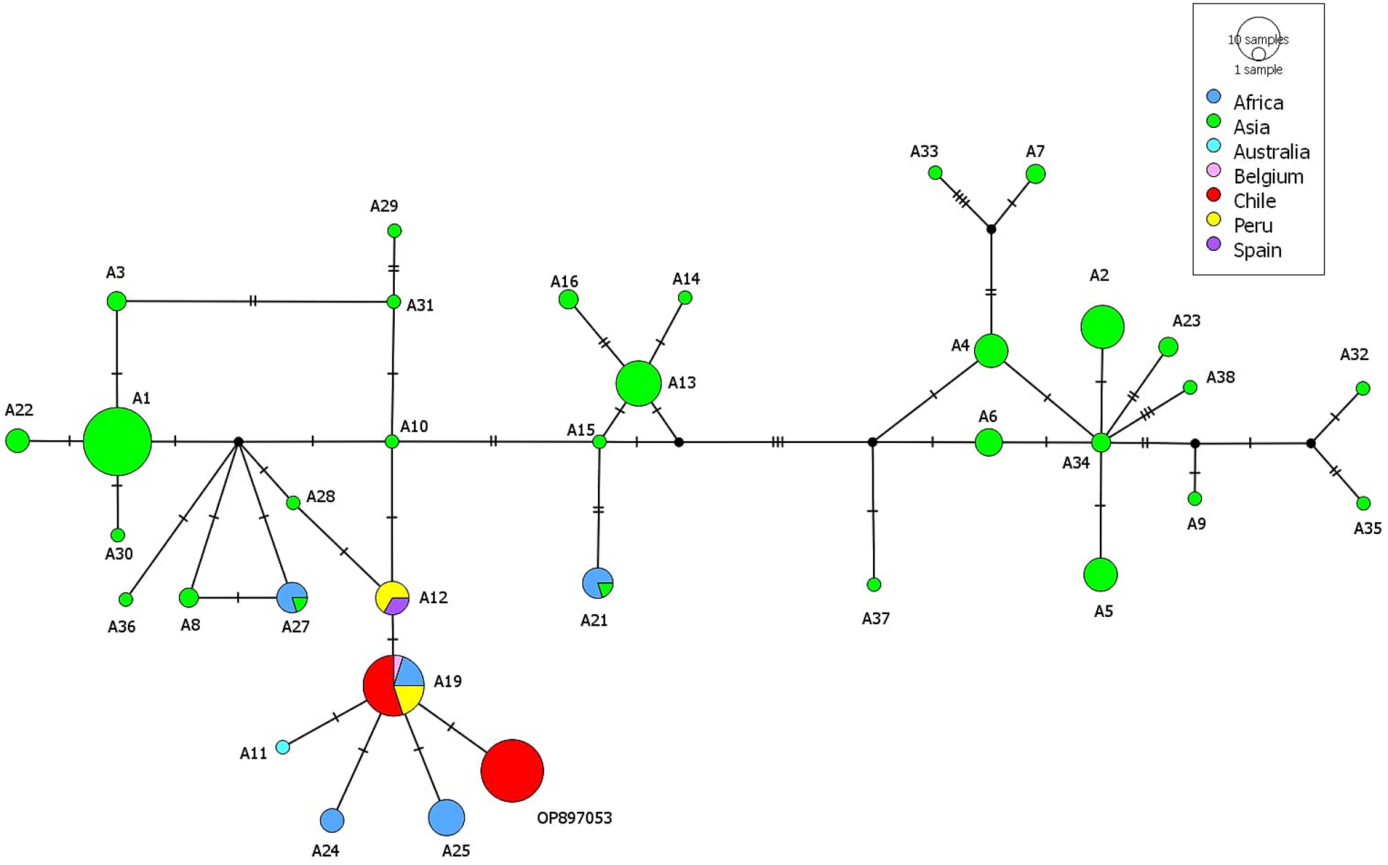
Haplotype network including the sequences acquired in the present study from *Hydatigera taeniaeformis* from black rats (*Rattus rattus*) and all similar *cox*1 sequences from GenBank identified as *H. taeniaeformis*.

Two haplotypes were found based on a 396 bp section of the *cox1*, differing in one nucleotide from each other. Hap1 (accession number OP897053) was found in 22 samples while 11 samples share 100% homology with the A19 haplotype previously reported in one sample from Belgium (isolate code: TtBMM) (9), four from Ethiopia (KT693060) (1) and in four samples from Peru (OQ569226-OQ569229) (3). The three sequences from Kodkod share 100% homology with Hap1 (OP897053). The haplotype network shows the largest green circles indicate that certain haplotypes are more prevalent or common, especially in samples from Asia. The sequences described in this study are closely related with haplotypes A24 and A25 described in Africa, A11 described in Australia and A12 described in Peru and Spain (Figure 3).

## 4 Discussion

Rats arrived to Europe from Asia during pre-Roman times 4,000–2,300 years BC (10) and then to the Americas with the Spanish Colonization (11). Domestic cats arrived to the Americas with the European colonization (4). Given that *H. taeniaeformis* requires both cats and rats to complete its life cycle, it is believed that this parasite was introduced to the Americas with the arrival of Europeans (1). In the case of North America, it is thought that the arrival of *Hydatigera* spp. may have occurred across the Holarctic region spreading from Eurasia into North America with their arvicoline intermediate hosts (12). The concurrent presence of small felids in more northern latitudes may have facilitated the expansion of *Hydatigera* spp. to North America. Under this hypothesis, the original wild definitive host in North America, prior to the arrival of domestic cats, would have been the bobcat (*Lynx rufus*). Wild felids in the Americas have acquired this parasite, as demonstrated in North American felids (12–14) as well as in the Southern Hemisphere, such as kodkods from central and southern Chile (15). In the case of intermediate host, *H*. *taeniaeformis* has been found to infect native rodents such as the Eastern deer mouse (*Peromyscus maniculatus*) from California (16), the Tuco-Tuco de Los Talas (*Ctenomys talarum*), a subterranean rodent in Argentina (17), wild rodents Yucatan deer mouse (*Peromyscus yucatanicus*), Gaumer’s spiny pocket mouse (*Heteromys gaumeri*), and Yucatán vesper mouse (*Otonyctomys phyllotis*) from Yucatan, Mexico (18) and the small-eared pygmy (*Oligoryzomys microtis*), the pygmy rice rats (*Oligoryzomys spp.*) and white-naped squirrels (*Simosciurus nebouxii*) in Peru (3).

The highest diversity of *H*. *taeniaeformis s.s.* described by Lavikainen (1) in East and South-East Asia with up to 35 *cox*1 haplotypes suggest that the species originates in this region. The low genetic diversity found in *H*. *taeniaeformis* in our study is expected since the relatively early arrival of the parasite to the Americas. Interestingly, only two haplotypes belonging to *H*. *taeniaeformis s*.*s*. were described in Europe including A12 from Spain and A19 from Belgium. Both haplotypes have been described to occur in Peru (3). A19 has also been described in East Africa (1) and in the present study. In the haplotype network A19 is closely related to A12, A11 (Australia), A24-A25 (South Africa) and OP897053 (this study). The genetic characterization component of this study uncovered two distinct haplotypes of *H*. *taeniaeformis* in the *R. rattus* populations examined from Southern Chile. The identification of a limited number of haplotypes suggests a relatively low genetic diversity within this region, which could be indicative of a recent colonization. Interestingly, one of the haplotypes (Hap1) is novel, with no identical matches in GenBank, suggesting potential regional adaptation or previously unrecorded genetic variation. In contrast, the second haplotype (A19) shows a wide geographical distribution, with matches in Africa, Europe, and other South American locations such as Peru.

The inclusion of Kodkods in this study is particularly noteworthy, as it provides rare genetic insights into the role of wild felids as definitive hosts for *H. taeniaeformis* in Southern Chile. The genetic analysis of proglottids extracted from road killed Kodkods revealed that they share the same haplotype (Hap1) identified in the black rat populations from southern Chile. This finding underscores the interconnectedness of wildlife and domestic species in the transmission dynamics of this parasite. Given the Kodkod’s status as a vulnerable species, understanding its role in the lifecycle of *H. taeniaeformis* not only contributes to our knowledge of parasitology but also has implications for conservation strategies.

The connection between Africa and Spain at the time of the colonization was based on logistical and economic reasons. The trips of the Spaniards commonly had a stopover in the Canary Islands where livestock was also brought to the Americas (19). Livestock from the Canary Islands most likely evolved from animals brought there from North Africa. In the case of *R. rattus*, a molecular characterization of populations from the Canary Islands detected a possible role of the islands as a faunal link with the American and European continents, suggesting that some rats that arrived in the Americas could have been originated not only in Europe but also in the archipelago (20). Therefore, we propose that the current population of *H*. *taeniaeformis* inhabiting South America descend from parasites from Europe but also from the Canary Islands and North Africa. Geographical clustering was evident, with distinct clusters of haplotypes from Africa, Australia, and Europe (including Belgium and Spain). Despite this, the presence of shared haplotypes across these regions suggests genetic exchange or a common ancestral lineage. Chile and Peru showed fewer haplotypes, indicative of reduced genetic diversity, potentially due to a founder effect or recent introduction of the parasite. A unique haplotype represented by a large, isolated red circle on maps of Chile suggests recent mutations or extended isolation from other populations.

During this study, the prevalence of *H. taeniaeformis* in black rats was determined to be 18.9% (33/174), which is consistent with findings from other regions that exhibit moderate to high rates of infection. For instance, this figure lies between the lower prevalence observed on Guafo Island in Chilean Patagonia (7%) (21) and the higher prevalence rates reported in Argentina (26.2%) (22) and certain areas of Mexico between 4.3% (23) and 28.2% (24). These variations underscore the influence of regional ecological dynamics, such as host population density, behaviour, and the ecosystem’s characteristics, on the transmission and maintenance of cestode infections. The comparative analysis with other geographical locations suggests that factors like climate, urbanization, and the effectiveness of local rodent control measures might contribute to the observed differences in prevalence rates. Moreover, our findings prompt further investigation into the life cycle of *H. taeniaeformis* within urban and peri-urban environments, particularly focusing on the potential zoonotic risks and the role of definitive feline hosts in the parasite’s dissemination.

Capture success remained consistent between 2022 and 2023, however, prevalence was higher in 2022, which might be due to environmental factors that we did not analyse. Additionally, a positive correlation was noted between body length and average parasite length (correlation coefficient = 0.40, *p* < 0.001), suggesting that longer animals tend to have longer parasites. A stronger correlation was observed between weight and parasite length (correlation coefficient = 0.76, *p* < 0.001), highlighting weight as a more significant predictor of parasite length.

Further analysis in our study also explored the correlation between body size of rats and the size of *H. taeniaeformis* metacestodes. In line with observations from other studies, our results indicate that bigger and older rats typically harbour larger metacestodes. Specifically, adult rats were found to host cestodes with an average length of 77.6 mm, which is significantly larger than those found in younger cohorts. This trend supports the prevailing view in parasitological research that the size of *H. taeniaeformis* metacestodes tends to increase as their rodent hosts age, likely due to prolonged exposure and accumulation over time. This finding underscores the potential influence of host age on the growth and development of the parasite, highlighting an important aspect of host–parasite dynamics.

In conclusion, this study provides new insights into the prevalence and genetic diversity of *H. taeniaeformis* in black rats in southern Chile. Our identification of limited genetic variability, including a novel haplotype, suggests localized adaptation and possibly recent introduction of this parasite into the region. The correlation between these genetic findings and historical human movements highlights the impact of past colonization and trade on the distribution of parasitic diseases. These results emphasize the need for ongoing research to have a better understanding of the transmission dynamics of *H. taeniaeformis*, which could aid in the development of targeted control strategies to reduce its impact on public and animal health.

## Data Availability

The datasets presented in this study can be found in online repositories. The names of the repository/repositories and accession number(s) can be found in the article/supplementary material.

## Footnotes

^1^ https://www.geneious.com

## References

[1] Lavikainen A Iwaki T Haukisalmi V Konyaev SV Casiraghi M Dokuchaev NE. Reappraisal of *Hydatigera taeniaeformis* (Batsch, 1786) (Cestoda: Taeniidae) *sensu lato* with description of *Hydatigera kamiyai* n. sp. Int J Parasitol. (2016) 46:361–74. doi: 10.1016/j.ijpara.2016.01.009, PMID: 26956060

[2] Deplazes P Eckert J Mathis A von Samson-Himmelstjerna G Zahner H. Parasitology in veterinary medicine Wageningen Academic Publishers (2016).

[3] Gomez-Puerta LA Vargas-Calla A Garcia-Leandro M Jara-Vila J Rojas-Anticona W Pacheco JI. Identification of wild rodents as intermediate hosts for *Hydatigera taeniaeformis* in Peru. Parasitol Res. (2023) 122:1915–21. doi: 10.1007/s00436-023-07892-6, PMID: 37272976

[4] Driscoll CA Clutton-Brock J Kitchener AC O’Brien SJ. The taming of the cat. Genetic and archaeological findings hint that wildcats became housecats earlier--and in a different place--than previously thought. Sci Am. (2009) 300:68–75. doi: 10.1038/scientificamerican0609-68 PMC5790555 19485091

[5] Bush AO Lafferty KD Lotz JM Shostak AW. Parasitology meets ecology on its own terms: Margolis et al. revisited. J Parasitol. (1997) 83:575–83. doi: 10.2307/3284227, PMID: 9267395

[6] Barton K. MuMIn: multi-model inference. Available at: http://r-forger-projectorg/projects/mumin/ (2009).

[7] Bowles J Blair D McManus DP. Genetic variants within the genus *Echinococcus* identified by mitochondrial DNA sequencing. Mol Biochem Parasitol. (1992) 54:165–73. PMID: 1435857 10.1016/0166-6851(92)90109-w

[8] Leigh JW Bryant D. Popart: full-feature software for haplotype network construction. Methods Ecol Evol. (2015) 6:1110–6. doi: 10.1111/2041-210X.12410

[9] Okamoto M Bessho Y Kamiya M Kurosawa T Horii T. Phylogenetic relationships within *Taenia taeniaeformis* variants and other taeniid cestodes inferred from the nucleotide sequence of the cytochrome c oxidase subunit I gene. Parasitol Res. (1995) 81:451–8. doi: 10.1007/BF00931785 7567901

[10] Wilson DE Reeder DAM. Mammal species of the world: A taxonomic and geographic reference Johns Hopkins University Press (2005).

[11] Otto GM Franklin CL Clifford CB. Biology and diseases of rats In: Fox JG Anderson LC Otto GM Pritchett-Corning KR Whary MT, editors. Laboratory animal medicine. Boston, MA: Academic Press (2015). 151–207.

[12] Hoberg EP Galbreath KE Cook JA Kutz SJ Polley L. Chapter 1 – Northern host–parasite assemblages: history and biogeography on the borderlands of episodic climate and environmental transition In: Rollinson D Hay SI, editors. Advances in parasitology. Boston, MA: Academic Press (2012). 1–97. 10.1016/B978-0-12-398457-9.00001-9 22726642

[13] Riser NW. The hooks of Taenioid Cestodes from North American felids. Am Midl Nat. (1956) 56:133–7. doi: 10.2307/2422449

[14] Mueller JF. Accessory suckers (?) in *Taenia taeniaeformis* from the California bobcat. J Parasitol. (1973) 59:562. doi: 10.2307/3278795, PMID: 4711672

[15] Acuña-Olea F Sacristán I Aguilar E García S López MJ Oyarzún-Ruiz P. Gastrointestinal and cardiorespiratory endoparasites in the wild felid guigna (*Leopardus guigna*) in Chile: richness increases with latitude and first records for the host species. Int J Parasitol Parasites Wildl. (2020) 13:13–21. doi: 10.1016/j.ijppaw.2020.07.013, PMID: 32793412 PMC7415641

[16] Theis JH Schwab RG. Seasonal prevalence of *Taenia taeniaeformis*: relationship to age, sex, reproduction and abundance of an intermediate host (*Peromyscus maniculatus*). J Wildl Dis. (1992) 28:42–50. doi: 10.7589/0090-3558-28.1.42, PMID: 1548801

[17] Rossin A Malizia AI Denegri GM. The role of the subterranean rodent *Ctenomys talarum* (Rodentia: Octodontidae) in the life cycle of *Taenia taeniaeformis* (Cestoda: Taeniidae) in urban environments. Vet Parasitol. (2004) 122:27–33. doi: 10.1016/j.vetpar.2004.03.001, PMID: 15158554

[18] Panti-May JA Hernández-Betancourt S Ruíz-Piña H Medina-Peralta S. Abundance and population parameters of commensal rodents present in rural households in Yucatan, Mexico. Int Biodeterior Biodegradation. (2012) 66:77–81. doi: 10.1016/j.ibiod.2011.10.006

[19] Rodero Serrano E Delgado JV Franganillo A. Primitive Andalusian livestock and their implication in the discovery of America. Arch Zootec. (1992) 41:383–400.

[20] López M Foronda P Feliu C Hernández M. Genetic characterization of black rat (*Rattus rattus*) of the Canary Islands: origin and colonization. Biol Invasions. (2013) 15:2367–72. doi: 10.1007/s10530-013-0466-3

[21] Seguel M Munoz F Paredes E Navarrete MJ Gottdenker NL. Pathological findings in wild rats (*Rattus rattus*) captured at Guafo Island, northern Chilean Patagonia. J Comp Pathol. (2017) 157:163–73. doi: 10.1016/j.jcpa.2017.07.006, PMID: 28942299

[22] Gómez Muñoz MA Robles M Milano A Navone GT. Helminth infection levels on *Rattus rattus* (Rodentia: Muridae) from Corrientes city, Argentina. Mastozool Neotrop. (2018) 25:221–7. doi: 10.31687/saremMN.18.25.1.0.18

[23] Panti-May JA Hernandez-Betancourt SF Rodriguez-Vivas RI Robles MR. Infection levels of intestinal helminths in two commensal rodent species from rural households in Yucatan, Mexico. J Helminthol. (2015) 89:42–8. doi: 10.1017/S0022149X13000576, PMID: 24000977

[24] Panti-May JA Palomo-Arjona EE Gurubel-Gonzalez YM Barrientos-Medina RC Digiani MC Robles MR. Patterns of helminth infections in *Rattus rattus* and *Mus musculus* from two Mayan communities in Mexico. J Helminthol. (2019) 94:e30. doi: 10.1017/S0022149X19000063 30714552

[25] Core Team. (2021). R: A language and environment for statistical computing. Vienna, Austria: R Foundation for Statistical Computing. Available at: https://www.R-project.org/

